# Miiuy Croaker Hepcidin Gene and Comparative Analyses Reveal Evidence for Positive Selection

**DOI:** 10.1371/journal.pone.0035449

**Published:** 2012-04-12

**Authors:** Tianjun Xu, Yuena Sun, Ge Shi, Rixin Wang

**Affiliations:** Laboratory for Marine Living Resources and Molecular Engineering, College of Marine Science, Zhejiang Ocean University, Zhoushan, People's Republic of China; Georgia Institute of Technology, United States of America

## Abstract

Hepcidin antimicrobial peptide (HAMP) is a small cysteine-rich peptide and a key molecule of the innate immune system against bacterial infections. Molecular cloning and genomic characterization of HAMP gene in the miiuy croaker (*Miichthys miiuy*) were reported in this study. The miiuy croaker HAMP was predicted to encode a prepropeptide of 99 amino acids, a tentative RX(K/R)R cleavage motif and eight characteristic cysteine residues were also identified. The gene organization is also similar to corresponding genes in mammals and fish consisting of three exons and two introns. Sequence polymorphism analysis showed that only two different sequences were identified and encoded two proteins in six individuals. As reported for most other species, the expression level was highest in liver and an up-regulation of transcription was seen in spleen, intestine and kidney examined at 24 h after injection of pathogenic bacteria, *Vibrio anguillarum*, the expression pattern implied that miiuy croaker HAMP is an important component of the first line defense against invading pathogens. In addition, we report on the underlying mechanism that maintains sequences diversity among fish and mammalian species, respectively. A series of site-model tests implemented in the CODEML program revealed that moderate positive Darwinian selection is likely to cause the molecular evolution in the fish HAMP2 genes and it also showed that the fish HAMP1 genes and HAMP2 genes under different selection pressures.

## Introduction

The innate immune system represents the first line of defense against invading pathogens. Antimicrobial peptides (AMPs) are an evolutionarily conserved component of the innate immune responses widely across all classes of life and play an important role in protecting organisms against microbial invasion by exhibiting strong antimicrobial activity [1], [2]. AMPs have been demonstrated to kill Gram negative and Gram positive bacteria, mycobacteria, enveloped viruses, fungi, and even transformed or cancerous cells [3]–[5]. According to secondary structure and amino acid sequence similarities, AMPs family contains three classes: *α*-helical structures, highly disulphide bonded (cysteine-rich) *β*-sheets, and those with a high percentage of proline or glycine residues [6], [7]. Thereinto, cysteine-rich antimicrobial peptides are important parts of the AMPs family and have been identified in the hemolymph of crustaceans and the fat bodies of insects [8].

Hepcidin antimicrobial peptide (HAMP), also named LEAP-1 (Liver-expressed antimicrobial peptide 1) is one kind of cysteine-rich AMPs and is a key molecule of the innate immune system against bacterial infections and in iron metabolism in organisms. HAMP have been identified only in vertebrates, including teleosts [3], [7], amphibians [3], birds [9], and mammals [10]. It is comprised of a signal peptide, a prodomain, and a mature peptide with six to eight conserved cysteine residues that form multiple disulfide bonds and stable *β*-sheets [11]. The mature peptide of HAMP comprise approximately 20–25 amino acids in length and it is reported to play a crucial role in increasing membrane permeability and subsequently resulting in a lethal effect on cellular pathogens. Mammals have only a single copy of the hepcidin gene (exception of the mice), while some fish have clusters of two or more copies of HAMP [11]–[13]. HAMP genomic organization is strongly conserved with three exons and two introns. Meanwhile, although the HAMP maintained high sequence similarities among different species, particularly for the conservative cysteine-rich domain, multiple variants have been found in many teleosts [7], [8], [14], [15]. Phylogenetic analysis based on the sequence clustering showed that HAMP of fish can be divided into two paralogus lineages, HAMP1 and HAMP2 [16]. HAMP1 is present in all fish species and is an orthologue of the mammalian sequences; HAMP2 sequences have been found only in acanthopterygians representing a more complex and diversified group [16]. In general, HAMPs have been detected mostly in liver but expression is also detected at lower levels in several other organs [17]–[19], however, in black rockfish, the hepcidin II was predominantly expressed in the liver and not detectable in other tissues [8]. There are a lot of strong evidences for HAMP antibacterial activity in fishes, in all cases, HAMP1 and HAMP2 genes activated their expression after bacterial or lipopolysaccharide (LPS) treatments [10], [12], [20], [21]. In general, positive selection leads to functional divergence of protein coding genes [22], [23]. Due to their direct interactions with molecules of pathogens, AMPs are reported to have evolved adaptively, it is likely that much of these variation results from positive selection on the ability to combat new or altered pathogens [24], [25]. Thus, HAMP genes may be an ideal choice to study adaptive molecular evolution in vertebrates.

Farming of miiuy croaker, *Miichthys miiuy*, is a growing industry in China. However, many bacterial and viral diseases especially occurred in juvenile miiuy croaker had caused reduced production and profits which have led to an increased interest in how the miiuy croaker protects itself against diseases. Little genetic information is available about innate immune response and the defense mechanisms that miiuy croaker displays against bacterial infections. In the present study, we report the identification of a hepcidin cDNA sequence and its gene organization and study the sequence diversity among different individuals. Gene expression analyses in different tissues after *Vibrio anguillarum* injection were performed to elucidate the possible role of hepcidin in response to resisting bacteria in miiuy croaker. Furthermore, to explore the evolutionary mechanisms of hepcidin in fish, studies of the molecular evolution of HAMP were discussed.

## Results

### cDNA and genomic characterization of miiuy croaker HAMP

**Figure 1:**
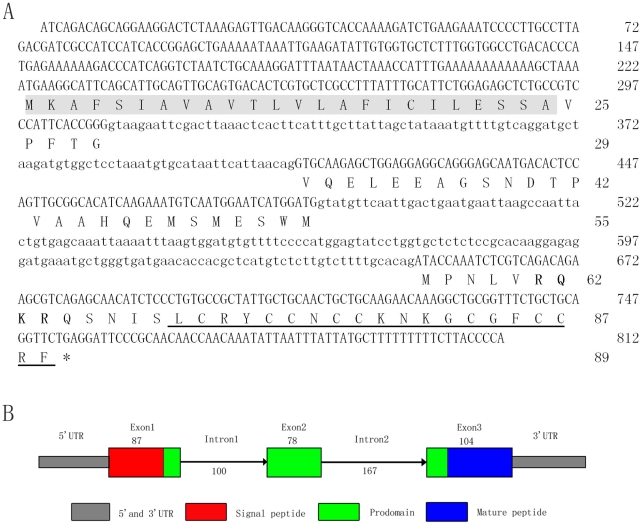
Genomic sequence (A) and schematic illustration (B) of miiuy croaker hepcidin gene. Exons are in uppercase and introns are in lowercase. An asterisk indicates the stop codon. The signal peptide and mature peptide is showed as gray background and underlined, respectively.

Full-length cDNA of hepcidin gene is 549 nucleotides (nt) that contained a 222 nt 5′ terminal untranslated region (UTR) followed by an open reading frame (ORF) of 270 nt encoded an 89 amino acid preprohepcidin, and 57 bp 3′ terminal partial UTR (GenBank accession No. HQ889844) (Fig. 1).

Position of cleavage of the signal peptide was predicted by PSORT (http://psort.hgc.jp/), and a signal peptidase cleavage site was detected between Ala^24^ and Val^25^ (Fig. S1). The typical HAMP domains (signal peptide, prodomain and mature peptide) were identified in the deduced amino acid sequence of the HAMP from miiuy croaker (Fig. 1) and typical RX(R/K)R motif of propeptide convertases was identified [26], and this conserved motif is composed of positively charged residues (Fig. S1). In addition, HAMP mature peptide contained the eight conserved cyseine residues.

Genomic DNA of hepcidin gene containing the intron-exon structure resulting in an 812 bp fragment is shown in Fig. 1. It was constituted by three exons and two introns. Exon 1 includes a 222 nt 5′ terminal UTR, the signal peptide and part of prodomain sequence following by intron 1 of 100 nt, exon 2 encoded only a part of the prodomain following by intron 2 of 163 nt, and exon 3 included the final part of the prodomain, complete mature peptide and 3′UTR. All of introns were flanked by the canonical “GT … AG” sequences. The genomic sequence is deposited in the GenBank with accession number HQ889846.

### Multiple alignment and phylogenetic analysis

Alignment and phylogenetic analysis of the amino acid sequences of the HAMPs from miiuy croaker and other vertebrates are shown in Fig. S1. Propeptide sequences alignment with different species show that HAMP, in particular the cysteine active region, is well conserved in all vertebrates. The HAMP sequences similarity among teleost is higher than that between fish and mammals. Miiuy croaker HAMP gene shares many of the same characteristics (e.g. conserved RX(R/K)R motifs) as other fishes. The deduced amino acid sequences of HAMP had 25.3–94.6% identity with those of mammal and teleost (Table S1).

**Figure 2:**
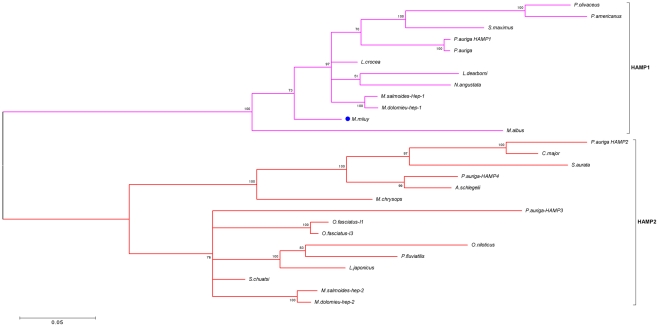
Phylogenetic tree of hepcidin gene from miiuy croaker and other teleost were constructed using Bayesian inference method.

To study the phylogenetic relationship of miiuy croaker HAMP gene and those of the other bony fish species, a phylogenetic tree was constructed using Bayesian inference in MrBayes3.1 software (Fig. 2). Phylogenetic analysis of different fish species HAMP sequences showed that these sequences clustered in two main groups: HAMP1 cluster containing acanthopterygian and non-acanthopterygian fish, and HAMP2 is present only in acanthopterygian fish (Fig. 2). Miiuy croaker HAMP gene belongs to the group HAMP1.

### Sequence diversity analysis

For analyzing polymorphism of HAMP gene in miiuy croaker, six individuals (fish A, B, C, D, E, and F) were used. An average of nine positive clones per individual was sequenced and total of 54 sequences were obtained. Only two different sequences were identified (Fig. S2), and two different isoforms that encoded different proteins were designated as Mimi-isoform 1 and Mimi-isoform 2 (HQ889844 and HQ889845). The fish A and F had two isoforms of HAMP gene, and fish B, C, D, and E only possessed Mimi-isoform 1 of HAMP gene The frequency of two isoforms was 72% (39/54), and 28% (15/54) in the 54 total sequences, respectively. Alignments revealed a high degree of sequence similarity between two sequences obtained (99.6%). Only two different isoforms were detected in six wild individuals indicated that low sequences variation existing in miiuy croaker hepcidin.

### Tissue Distribution and Expression analysis after bacterial infection

**Figure 3:**
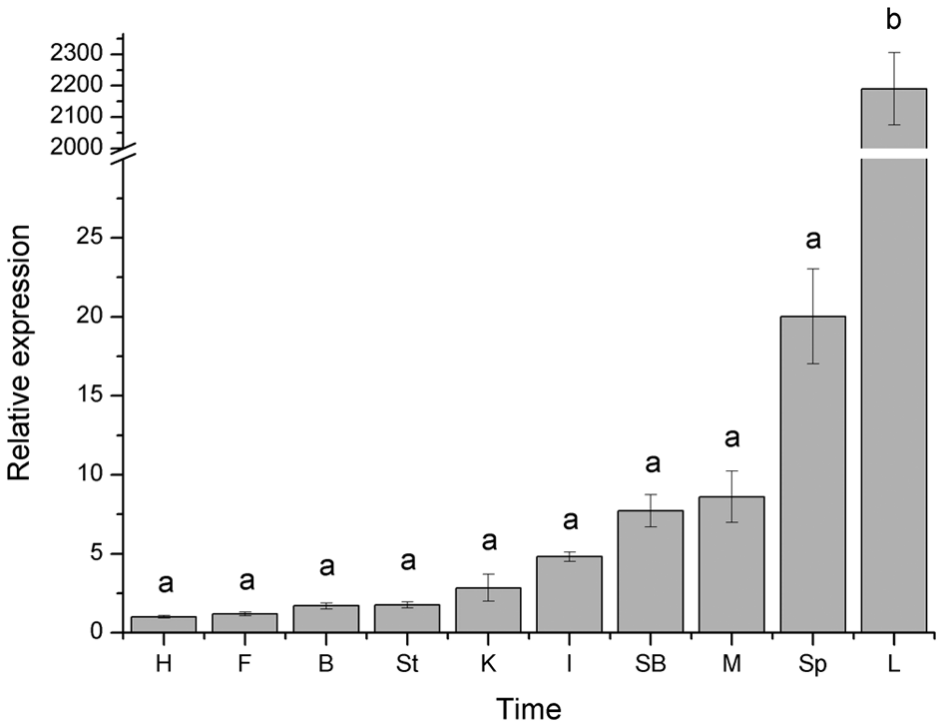
Expression of hepcidin gene in various tissues (heart (H), fin (F), brain (B), stomach (St), kidney (K), intestines (I), Swim bladder (SB), muscle (M), spleen (Sp), and liver (L) of uninfected miiuy croaker. Values with the same superscript are not significantly different (*P*>0.05).

In order to establish the expression patterns of the HAMP transcripts in tissues, mRNA were quantified in a wide range of tissues from miiuy croaker (Fig. 3). Real-time quantitative RT-PCR demonstrated that HAMP gene was ubiquitously expressed in ten tissues, but the expression level was distinctly different. The highest expression was observed in the liver, followed by moderate expression in spleen, muscle and swim bladder, and low expression in other tissues.

**Figure 4:**
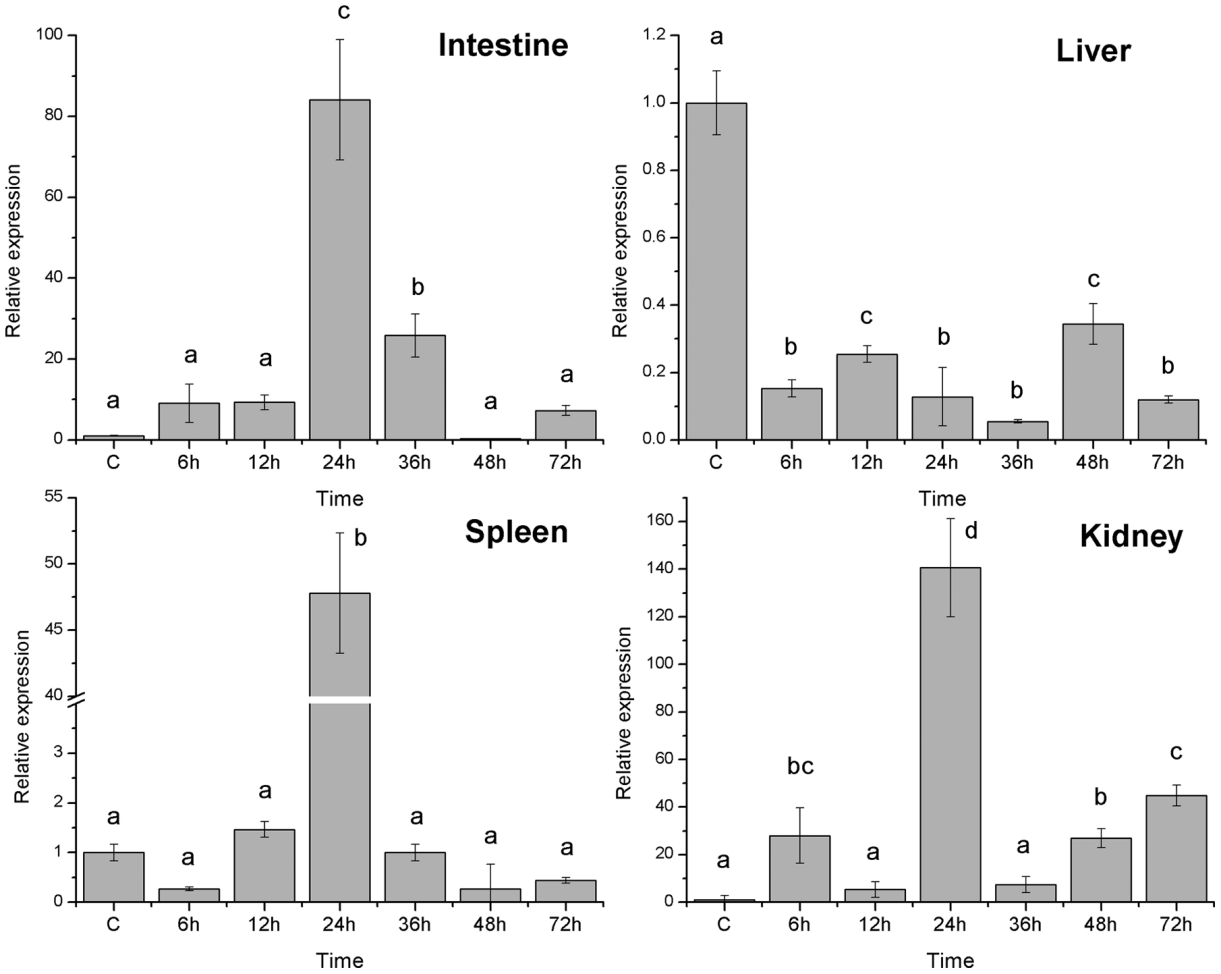
Expression of hepcidin gene in four tissues after injection with *V. anguillarum* sampled at six time point. Values with the same superscript are not significantly different (*P*>0.05).

Challenge of miiuy croaker with the pathogenic bacteria, *V. anguillarum*, resulted in significant changes in the expression of HAMP from infection starting time to 72 hours (h) after challenge in four important immune organs (Fig. 4). In liver, the expression level of HAMP gene sustained decreased from infection starting time to 72 h after challenge; the lowest expression level was checked at 36 h. In spleen, kidney and intestine, the expression level of HAMP gene significant increased from 12 h to 24 h (*P*<0.05), the high expression level were checked at 24 h after infection, following by a recovery to normal level after 72 h except for kidney.

### Molecular evolution analyses

Due to high sequence divergence between the two groups of fish HAMP genes was observed (Fig. S1 and Fig. 2), HAMP1 sequences group, HAMP2 sequences group and mammalians HAMP group were used for evolutionary analyses, respectively. The random sites models demonstrate extreme variability in selective pressure among sites. In HAMP1 group, model M3 appears to be a better fit to the data than the M0 model, suggesting variable selection pressure across the HAMP1 sequences. But the alternative hypothesis of M2 and M8 models were not significantly better than the null hypothesis models (M2 vs M1, *p* = 1.00; M8 vs M7, *p* = 1.00) (Table 1). Therefore, no reliable positive selection sites were detected in fish HAMP1 group. In fish HAMP2 group, M3 model also appears to be a better fit to the data than the M0 model, and the alternative hypothesis model (M8) was significantly better than the null hypothesis model (M7). The site-specific analysis in PAML identified many positively selected sites in the HAMP2 group (Fig. S3 and Table 1). Twenty-seven positively selected sites were detected under M8 model including four sites (position 67, 87, 88, and 94) which *p*>0.95, Posterior probabilities for site classes have been calculated by Bayes empirical Bayes (BEB) [27]. Like the fish HAMP1 group, these alternative hypothesis models were not significantly better than the null hypothesis models (M2 vs M1, *p* = 0.88; M8 vs M7, *p* = 0.47), and there were no positively selected sites detected in mammalians HAMP group (Table 2).

**Table 1:** Evidence of positive Darwinian selection from site-specific model analyses for the HAMP gene of fish.

Groups	Model	*P*	Log-li*k*elihood	Model comparison	Parameter estimates	Positively selected sites
Fish HAMP1	M0 (One ration)	1	−1044.353		*K* = 3.332,*ω* = 0.413	None
	M3 (discrete)	5	−1025.981	M3 vs M0, 2ΔLnL = 36.74, df = 4, *p* = 1.0E-6	*K* = 3.585, *p*_0_ = 0.517, *p*_1_ = 0.171, *p*_2_ = 0.311,*ω*_0_ = 0.023,*ω*_1_ = 0.948,*ω*_2_ = 0.948	Not analyzed
	M1a (Nearly Neutral)	1	−1026.006		*K* = 3.619, *p*_0_ = 0.527, *p*_1_ = 0. 473,*ω*_0_ = 0.028,*ω*_1_ = 1.0	Not allowed
	M2a (Positive Selection)	3	−1026.006	M2 vs M1, 2ΔLnL = 0, df = 2, *p* = 1.00	*K* = 3.619, *p*_0_ = 0.527, *p*_1_ = 0.354, *p*_2_ = 0.119,*ω*_0_ = 0.027,*ω*_1_ = 1.0,*ω*_2_ = 1.0	
	M7 (beta)	2	−1026.031		*K* = 3.582, *P* = 0.075, *q* = 0.084	Not allowed
	M8 (beta and omega)	4	−1026.026	M8 vs M7, 2ΔLnL = 0.01, df = 2, *p* = 1.00	*K* = 3.592, *P*_0_ = 0.581, *p*_1_ = 0.419, *p* = 0.101,*q* = 0.914,*ω* = 1.0	
Fish HAMP2	M0 (One ration)	1	−1117.794		*K* = 2.53,*ω* = 0.572	None
	M3 (discrete)	5	−1086.262	M3 vs M0, 2ΔLnL = 63.06, df = 4, *p* = 0.00	*K* = 2.525, *p*_0_ = 0.602, *p*_1_ = 0.177, *p*_2_ = 0.253,*ω*_0_ = 0.101*ω*_1_ = 1.598, *ω*_2_ = 1.598	Not analyzed
	M1a (Nearly Neutral)	1	−1088.879		*K* = 2.403, *p*_0_ = 0.552, *p*_1_ = 0.448,*ω*_0_ = 0.062, *ω*_1_ = 1.0	Not allowed
	M2a (Positive Selection)	3	−1086.262	M2 vs M1, 2ΔLnL = 5.23, df = 2, *p* = 0.07	*K* = 2.585, *p*_0_ = 0.602, *p*_1_ = 0, *p*_2_ = 0.398,*ω*_0_ = 0.101,*ω*_1_ = 1,*ω*_2_ = 1.598	
	M7 (beta)	2	−1090.654		*K* = 2.428, *P* = 0.109, *q* = 0.112	Not allowed
	M8 (beta and omega)	4	−1086.276	M8 vs M7, 2ΔLnL = 8.76, df = 2, *p* = 0.01	*K* = 2.586, *P*_0_ = 0.604, *p*_1_ = 0.396, *p* = 11.360, *q* = 99.000,*ω* = 1.602	67, 87, 88, 94

**Note:** *P* number of parameters in the *ω* distribution, *K* estimated transition/transversion rate ration, *ω* selection parameter, and *p*_n_ proportion of sites that fall into the *ω_n_* site class. *p*, *q* shape parameters of the β function (for models M7 and M8). Only positively selected sites with posterior probability >0.95 are shown.

**Table 2:** Evidence of positive Darwinian selection from site-specific model analyses for the HAMP gene of mammalians.

	1	−989.958		*K* = 2.090,*ω* = 0.357	None
M3 (discrete)	5	−962.409	M3 vs M0, 2ΔLnL = 55.10, df = 4, *p* = 0.00	*K* = 2.375, *p*_0_ = 0.467, *p*_1_ = 0.185, *p*_2_ = 0.348,*ω*_0_ = 0.043*ω*_1_ = 0.459,*ω*_2_ = 1.303	Not analyzed
M1a (Nearly Neutral)	1	−962.584		*K* = 2.317, *p*_0_ = 0.526, *p*_1_ = 0.474,*ω*_0_ = 0.056,*ω*_1_ = 1.0	Not allowed
M2a (Positive Selection)	3	−962.462	M2 vs M1, 2ΔLnL = 0.24, df = 2, *p* = 0.88	*K* = 2.374, *p*_0_ = 0.531, *p*_1_ = 0.381, *p*_2_ = 0.087,*ω*_0_ = 0.061,*ω*_1_ = 1,*ω*_2_ = 1.664	M0 (One ration)
M7 (beta)	2	−963.172		*K* = 2.284, *P* = 0.180, *q* = 0.206	Not allowed
M8 (beta and omega)	4	−962.411	M8 vs M7, 2ΔLnL = 1.52, df = 2, *p* = 0.47	*K* = 2.369, *P*_0_ = 0.623, *p*_1_ = 0.377, *p* = 0.469, *q* = 2.968,*ω* = 1.258	

**Note:** *P* number of parameters in the *ω* distribution, *K* estimated transition/transversion rate ration, *ω* selection parameter, and *p*_n_ proportion of sites that fall into the *ω_n_* site class. *p*, *q* shape parameters of the β function (for models M7 and M8).

## Discussion

HAMP genes are significant elements of the innate immunity in vertebrates; they have been attracting attention of many scientists. HAMPs are important cysteine-rich bioactive peptides of the innate immune system in vertebrates including teleosts, amphibians, birds, and mammals. To date, an increasing number of HAMP genes have been found in human, house mouse, and other higher vertebrates [16]. So far, HAMP genes were described in some teleost [6], [28]. However, at present, only a few fish species has been make out with a comprehensive and systematic study. In the present study, we have identified HAMP cDNA sequence and genomics organization, and analyzed its sequence diversity, expression pattern in miiuy croaker, and discussed the molecular evolution pattern.Miiuy croaker HAMP cDNA was found to have an open reading frame of 270 nt, encoding for a putative 89 amino acid peptide that shares many characteristic features with HAMPs from other species. The putative HAMP prepeptide of miiuy croaker shows similarity with other HAMPs throughout the entire length, and possess highest identity in the mature peptide region (Fig. S1). Comparison with other sequences, the miiuy croaker HAMP peptide is predicted to have two different cleavage sites, thus dividing it into three regions: signal peptide, prodomain and mature peptide. From Fig. S1, we can see that two putative cleavage sites observed in miiuy croaker are similar to those found in other species. The conserved eight cysteine sites involved in one vicinal and three interstrand disulfide bridges [29], shared for all of the teleost and mammalians since this cysteine-rich structure of AMPs is known to confer antimicrobial activity to the protein [17], it can be expected that miiuy croaker HAMP will possess this immunological functionality. The conserved RX(R/K)R cleavage site motifs for the propetide convertase furin known to cleave propeptide [30], [31], were discovered in almost all fish species except for *P. auriga* and *C. major*, this motif is composed of positively charged residues. The putative mature peptide includes cationic and hydrophobic amino acids showed that this HAMP could adapt an anphipathic structure and other peptides displaying antimicrobial activities [32], [33].

Compared to the corresponding genomics reported for many fish HAMP genes (Table S2), intron 1 were ranging between 87 and 114 nt in length, and intron 2 were ranging between 118 and 191 nt in length. Miiuy croaker HAMP gene has a similar organization as the corresponding genes in mammals and other fish species, consisting of three exons and two introns, although the lengths of introns and exons differ [15], [28], [33], [34]. All fish HAMP possessed an 87 nt exon 1 in length, a 78 nt exon2 in length except for *M. chrysops*, *O. fasciatus* isoform 1 and *O. niloticus*. However, there were big variations of exon 3 in length among all fish species. Length difference of prepeptides is mainly reflected in the prodomain region. In general, the length of intron 2 is greater than intron 1 in all studied fish species.

Many studies showed that HAMP as an AMP gene has an unusual expression pattern given that it is highly expressed in the liver. In this study, as seen in mammals and many fish species [7], [11], [14], [15], [17], [18], [20], [34], the level of HAMP expression in miiuy croaker was highest in liver (*P*<0.05); meanwhile, HAMP mRNA has been detected in all assay tissues. Many fish species discovered highest HAMP expression levels in the liver, but higher levels are often found in other tissues as well [15], [33]. In miiuy croaker, spleen, muscle and swim bladder are also showed the higher expression levels. In general, this tissue expression of HAMP gene differs between different fish species [7], [8], [12], . In order to elucidate the function of miiuy croaker HAMP, we analyzed the levels of hepcidin transcription under bacteria infection. As expected, challenging miiuy croaker with pathogenic bacteria, *V. anguillarum*, significantly up-regulated the HAMP expression in spleen, intestine and kidney. At 24 h post injection, hepcidin transcript was strongly induced in the spleen, intestine and kidney. After one day, the transcript was highly decreased (*P*<0.05). Expression levels of HAMP gene were found first up-regulated and then down-regulated, and finally recovery to normal level throughout the infection process suggest that crucial interference of cellular function occurs under a semilethal concentration of pathogenic bacteria in immune tissues. If the infected concentration of pathogenic bacteria is greater than semilethal, the cellular function of immunity organs may be destroyed, and the corresponding expression may decrease [35]. Interestingly, the expression level of HAMP gene sustained decreased from infection starting time to 72 h after challenge in the miiuy croaker liver. It has been reported that the response of HAMP genes in other fish species to pathogenic bacteria challenge resulted in a up-regulated expression in the liver [8], [14], [15], [28], these results indicated that different fish species have different expression patterns of HAMP genes. In addition, HAMP genes expression pattern also showed the difference between HAMP1 gene and HAMP2 gene and between different types of HAMP gene in fish [8], [28]. These results in this study implied that hepcidin plays an important role in the immune response of miiuy croaker to infection.

Hilton and Lambert [16] had shown that the fish HAMPs can be divided into two paralogous lineages referred to as HAMP1 and HAMP2, HAMP1 is present in every fish species and is an orthologue of the mammalian HAMP gene, HAMP2 genes were detected only in acanthopterygians. Based on the sequences clustering, suggested by phylogenetic tree, these fish paralogue HAMP genes separates into two main different groups with HAMP1 and HAMP2. Miiuy croaker is a non-acanthopterygian species, HAMP2 gene should not be found, thus, its HAMP gene belongs to the HAMP1 group. Phylogenetic tree clearly confirmed that this classification is accurate.

Unlike other vertebrates, some fish species have multiple hepcidin homologues [11]–[13]. Due to their direct interactions with molecules of pathogens, AMPs are reported to have evolved adaptively through an accelerated rate of amino acid substitutions to combat new or altered pathogens [24], [25]. It suggested that multiple copies related AMPs sequences have evolved by gene duplication, the rapid functional divergence among these copies could be associated with an accelerated rate of amino acid substitutions among the duplicated genes [6], [27], [36]. Gene duplications represent a major evolutionary force in vertebrate organisms, and most gene duplicates are lost or silenced during evolution [37], [38]. In history, three rounds of large-scale gene duplications (1R, 2R and 3R or fish-specific genome duplication) have been identified in vertebrates [26], [39], and phylogenetic analysis showed that the HAMP2 group would have appeared after the 3R genome duplication [28]. The acanthopterygians appear to have at least two HAMP homologues, meanwhile, non-acanthopterygians retaining only the HAMP1 gene due to most duplicates (HAMP2) have been lost. HAMP1 is present in every fish species and is considered as the orthologue of the mammalian hepcidin sequences. In contrast, HAMP2 paralogs gene have been found only in acanthopterygian fish [16].

The functional divergence of duplicated genes occurs through three main processes: neofunctionalization, subfunctionalization and subneofunctionalization [40]–[42]. In these processes, the positive selection plays a crucial role in accelerating the fixation of advantageous mutations [28]. Fixation of HAMP2 genes in acanthopterygian fish could be favored by the radication of teleosts in different marine and brackish environments and the operation of positive Darwinian selection [6], [28].

Accelerated rate of amino acid substitutions among duplicated genes is the main indication of adaptive evolution [25], identifying genes that have evolved by adaptation is central to understanding the pattern of molecular evolution. HAMP1 and HAMP2 groups may have different ways of molecular evolution under different selection pressures. In contract with major histocompatibility complex genes, there is little direct evidence for positive selection on AMPs [25]. To study the molecular evolution of the HAMP1 and HAMP2 sequences, we conducted analysis of positive selection using a series of models. If the ratio of nonsynonymous nucleotide differences (*d_N_*) to synonymous nucleotide differences (*d_S_*) between sequences is significantly greater than one, positive selection can be inferred [43], [44]. The *d_N_*/*d_S_* (*ω*) ratio is greater than one for some AMPs in both vertebrates and invertebrates [25]. In this study, the average *ω* ratio for comparisons between fish HAMP1 and HAMP2 group, and mammalians HAMP group show that *ω*<1, indicated that HAMP genes were under purifying selection that is probably due to function constrains [45], this shows that a disproportionately higher than number of changes occur at synonymous sites than expected by chance. This result is consistent with previous studies of fish AMPs genes including hepcidin gene and piscidin gene [6], [46].

Model 3 appears to be a better fit to the data than the M0 model, suggesting variable selection pressure across the fish HAMP1, fish HAMP2 and mammalian HAMP sequences. Cysteines are highly conserved in hepcidin gene are not under positive selection, but other sites near their mature peptide region are frequently positively selected [25]. Despite the high sequences similarity and the presence of eight conserved cysteine residues in the mature peptide region, site-specific analysis showed that many of the codons in the mature peptide regions of HAMP2 are subjected to positive Darwinian selection, however, no codons of fish HAMP1 and mammalian HAMP mature peptide region were under positive selection, site-specific analyses for fish HAMP1 and mammalian HAMP showed no evidence of positive Darwinian selection (Table 1, Table 2). Similar molecular evolution pattern of fish HAMP1 and mammalian HAMP suggested that the fish HAMP1 is an orthologue of the mammalian HAMP gene. HAMP2 paralogs genes is only in acanthopterygian fish could be favored by the radication of teleosts in different marine and brackish environments and the operation of positive Darwinian selection [6], [28]. The differential pattern of molecular evolution in fish HAMP1, HAMP2 and mammalian HAMP could be associated with their specific habitats and surrounding pathogens. Evolution divergence might have caused differences in immunological function of these peptides between these different species [6]. Some positive selection sites were detected suggest important physiology roles for HAMP2, undertaking functional analyses to determine whether these sites played a crucial role in the adaptive evolution of HAMP genes might be useful.

With fish HAMP1 and mammalian HAMP different, four positively selected amino acids (67, 87, 88, and 94) with posterior probabilities greater than 0.95 were detected in fish HAMP2 sequences that might have evolved under moderate positive Darwinian selection. In addition, like the conclusion obtained from Padhi et al. [6], site-specific analyses for mammalian hepcidins also showed no evidence of positive Darwinian selection. As a whole, the fish HAMP1 and mammalian HAMP sequences have experienced purifying selection, but the fish HAMP2 might have evolved under moderate positive Darwinian selection.

## Materials and Methods

### Ethics statement

All work was conducted with the approval of the Animal Ethics Committee.

### Challenge and Tissue Collection

Two-years-old adult miiuy croakers were obtained from Zhoushan Fisheries Research Institute (Zhejiang, China). Only healthy fish, as determined by general appearance and level of activity, total twenty-two fishes were used in this study. Ten tissues of uninfected miiuy croaker were removed and kept at −80°C until RNA and DNA were extracted. Challenge of miiuy croaker with *V. anguillarum* was performed as described by Xu et al. [47]. Fish were anaesthetized by immersion in MS222 and injected intraperitoneally with 1 ml bacteria suspension (3.0×10^7^ CFU/ml). Control fish injected with phosphate-buffered saline were maintained in separate tanks. The infected and health fish were killed at 6 h, 12 h, 24 h, 36 h, 48 h, and 72 h after injection, respectively. Four tissues (liver, spleen, intestine, and kidney) were removed and kept at −80°C until use. Immediately following tissue excision, samples were placed into 1 mL of Trizol reagent and homogenised.

### EST Analysis and cDNA cloning

In our library, anormalized cDNA library was constructed from the spleen of miiuy croaker. A total of 5053 ESTs from the library were sequenced and compared with sequences in the GenBank database. BLAST analysis of all of the EST sequences showed that one EST sequences (GW670551) [48], similar to hepcidin in *Pseudosciaena crocea* and other fish species. To isolate full length cDNA of hepcidin gene, this EST clone was separately sequenced from both forward and reverse directions with vector primers M13F and M13R, and sequencing was repeated for three times. The full-length cDNA of clone was obtained by overlapping the forward and reverse strand sequences.

### Genomic Cloning and Sequence Polymorphism Analysis

To identify hepcidin gene genomic organization of miiuy croaker, the primer pair Mimi-H-F (5′-GTGCTCTTTGGTGGCCTG-3′) and Mimi-H-R (5′-TTGTTGGTTGTTGCGGGA-3′) which located on 5′ and 3′ untranslated region (UTR) respectively, was designed to amplify all introns and exons of hepcidin gene. Exon-intron junctions were deduced according to the known homologous sequences of the other vertebrates. In addition, to investigate the polymorphism of hepcidin gene, the primer pair ORF-F (5′-TGTGGTGCTCTTTGGTGGC-3′) and ORF-R (5′-TATTTGTTGGTTGTTGCGG-3′) was used for amplifying the complete open reading frame (ORF) sequences from cDNA templates of six captured wild individuals. These PCR products were resolved by electrophoresis on 1.5% agarose gels and the fragments of interest were excised, and then purified using the Gel Extraction Kit (Takara). The purified fragments were ligated into PMD-19T vectors (Takara) and cloned to TOP10 cells according to the standard protocol [49]. Positive clones were screened via PCR with M13+/- primers. At least three clones were sequenced per fragment using the ABI 3730xl automated sequencer with M13 primer.

### RT-qPCR Analysis of mRNA expression

The mRNA expression patterns of hepcidin gene in different tissues (liver, spleen, kidney, intestines, heart, muscle, stomach, brain, swim bladder, and fin) of healthy miiuy croakers and in four tissues (liver, spleen, kidney, and intestines) of infected and health miiuy croakers were determined using real-time RT-PCR. Tissue samples from three individuals were mixed for RNA preparation. Primers HAMP-RT-F (5′-TCGTGCTCGCCTTTATTTG-3′) and HAMP-RT-R (5′-AACCGCAGCCTTTGTTCTT-3′) were used for amplifying gene fragment. Real-time quantitative PCR was conducted on a 7500 Real-time PCR system (Applied Biosystems, USA). Amplifications were carried out at a final volume of 20 µl, containing 1 µl cDNA sample, 10 µl SYBR Green Real-time PCR master mixtures (Takara), 0.4 µl ROX II, 1 µl of each primer and 6.6 µl ddH_2_O. The reaction carried out without the template was used as blank control. PCR amplification was performed in triplicate wells, using the following conditions: 10 sec at 95°C, followed by 40 cycles consisting of 5 sec at 95°C and 34 sec at 60°C, dissociation curve analysis was performed after each assay to determine target specificity. Expression of β-actin was used as internal control in gene expression analysis. The primers β-actin-RT-F and β-actin-RT-R [50] were used for RT-PCR of β-actin expression. Comparisons between groups were made by one-way analysis of variance followed by a Duncan test for identification of the statistically distinct groups.

### Sequence alignment and phylogenetic analysis

Multiple alignments of the HAMP sequences from different species were performed using the Muscle [51]. The appropriate model of sequences evolution was determined with jModeltest [52] under Bayesian Information Criterion (BIC). The GTR+I+G model of sequence evolution for phylogenetic analysis. Phylogenetic tree was reconstrucred using Bayesian inference in MrBayes3.1 [53]. The program was run with 5,000,000 generations with a burn-in of 25%.

### Test for selection

Phylogenetic analysis showed that HAMP of fish can be divided into two paralogus lineages (HAMP1 and HAMP2), therein, HAMP1 is an orthologue of the mammalian sequences; paralogs gene (HAMP2) is generated in 3R genome replication [16.28]. Closely related genes show rapid functional diversification immediately after a duplication event until they have adapted to their new function [25]. A total of twenty-seven unique sequences representing twelve HAMP1 sequences and fifteen HAMP2 sequences, respectively, were used for evolutionary analyses.

In order to estimate the selective constraints on the HAMP gene, evolutionary analysis were performed with PAML 4.5 program suite [54], [55]. The hypothesis of positive selection was tested using site-specific model in the CODEML program. We employed the random-sites models [56] assuming several heterogeneous sites with different *ω* parameters without a priori knowledge of which class (neutral, purifying, or positive selection) a given codon belongs to. To account whether positive selection has been operating on any codon sites, we estimated parameters under six different codon substitution models (M0, M1a, M2a, M3, M7, and M8 model) [56]. The likelihood ratio tests (LRT) were performed to compare the corresponding models with and without selection (ie, M0 vs M3, M1a vs M2a, and M7 vs M8). When the alternative models M2a and M8 suggest the presence of codons with *ω*>1, this can be considered as evidence of positive selection [56]. Posterior probabilities for site classes have been calculated by Bayes empirical Bayes (BEB) in the case of models M2a and M8 [27]. Statistical significance is determined by comparing twice the log-likelihood difference (2ΔLnL) to a χ2 distribution with degrees equal to the difference in the number of parameters between the models to be compared [54]. The posterior means of *ω* for some sites classes are great than one (calculated are the average of *ω* over all sites classes weighted by the posterior probabilities), those sites are likely to be under positive selection [27].

## Supporting Information

Figure S1 **Alignment of deduced amino acid sequences of the miiuy croaker hepcidin gene with those of other species.** Gaps used to maximize the alignment are shown by dashes. Conserved cysteins are shown in black background, the box indicates RX(K/R)R cleavage motifs.
(JPG)

Figure S2 **Nucleotide sequences and amino acid sequences for hepcidin alleles of miiuy croaker.** Dots indicate identity with the top sequences.
(JPG)

Figure S3 **Amino acid sequence comparison among fish HAMP2 sequences.** Positively selected sites identified using M8 model (Table 1) are shaded in black background.
(JPG)

Table S1 **Percent identity of amino acid** (**aa**) **sequences calculated versus the miiuy croaker HAMP sequence.**
(DOC)

Table S2 **Genomic organization of hepcidin genes of different fish species.**
(DOC)
