# Supplementary figures and images for: Miiuy Croaker Hepcidin Gene and Comparative Analyses Reveal Evidence for Positive Selection
Source: PLoS One. 2012 Apr 12;7(4):e35449. doi: 10.1371/journal.pone.0035449 (PMC3325200; doi:10.1371/journal.pone.0035449)

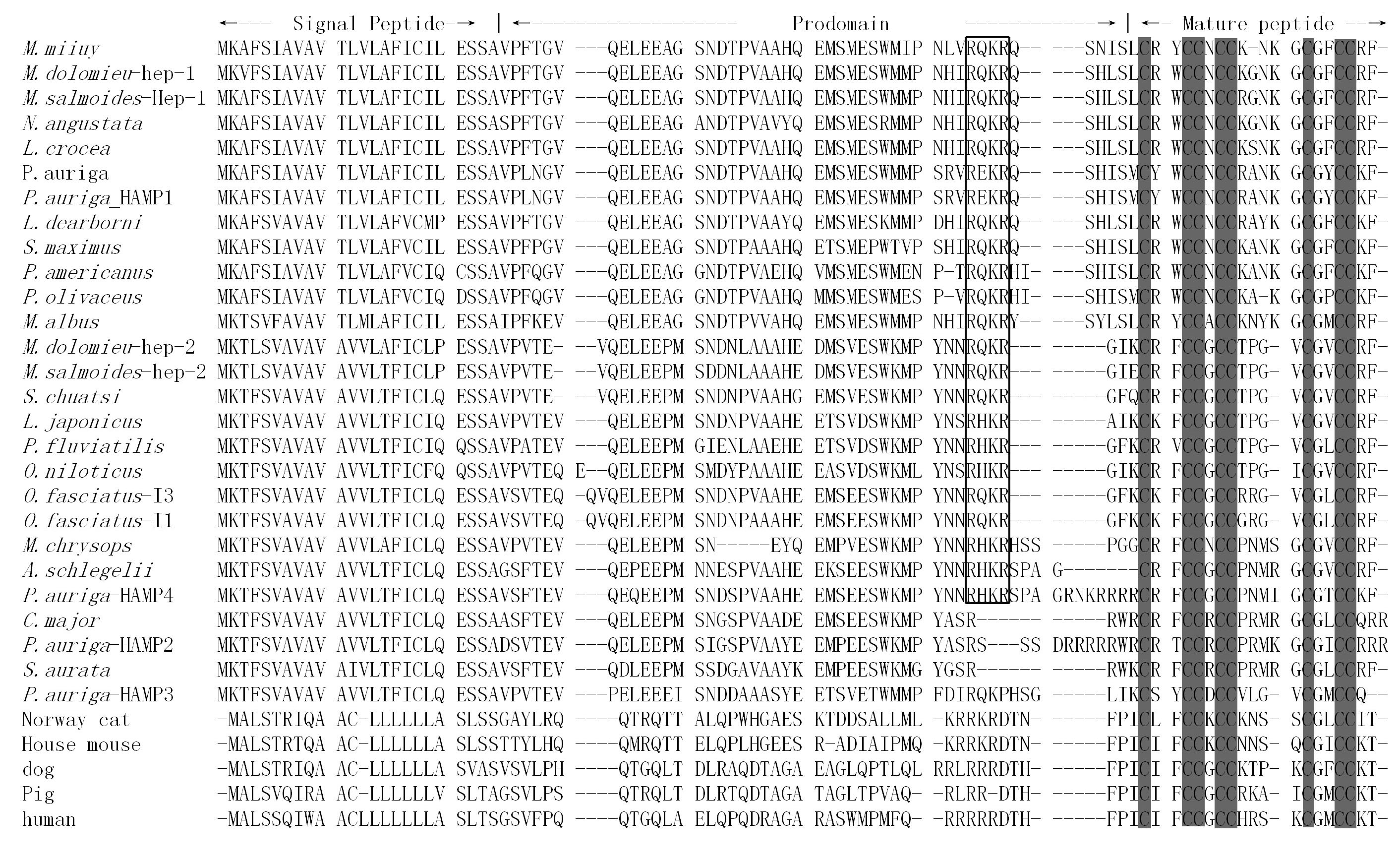

Supplement: Figure S1 — Alignment of deduced amino acid sequences of the miiuy croaker hepcidin gene with those of other species. Gaps used to maximize the alignment are shown by dashes. Conserved cysteins are shown in black background, the box indicates RX(K/R)R cleavage motifs. (JPG) [file pone.0035449.s001.jpg]

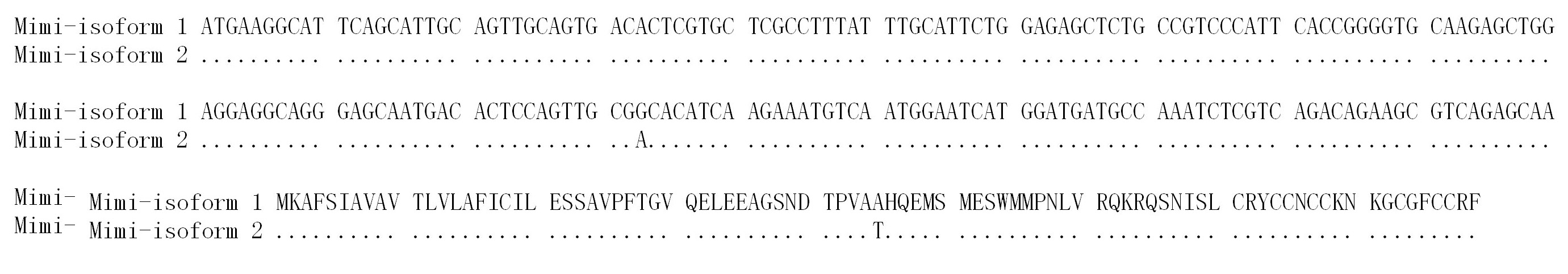

Supplement: Figure S2 — Nucleotide sequences and amino acid sequences for hepcidin alleles of miiuy croaker. Dots indicate identity with the top sequences. (JPG) [file pone.0035449.s002.jpg]

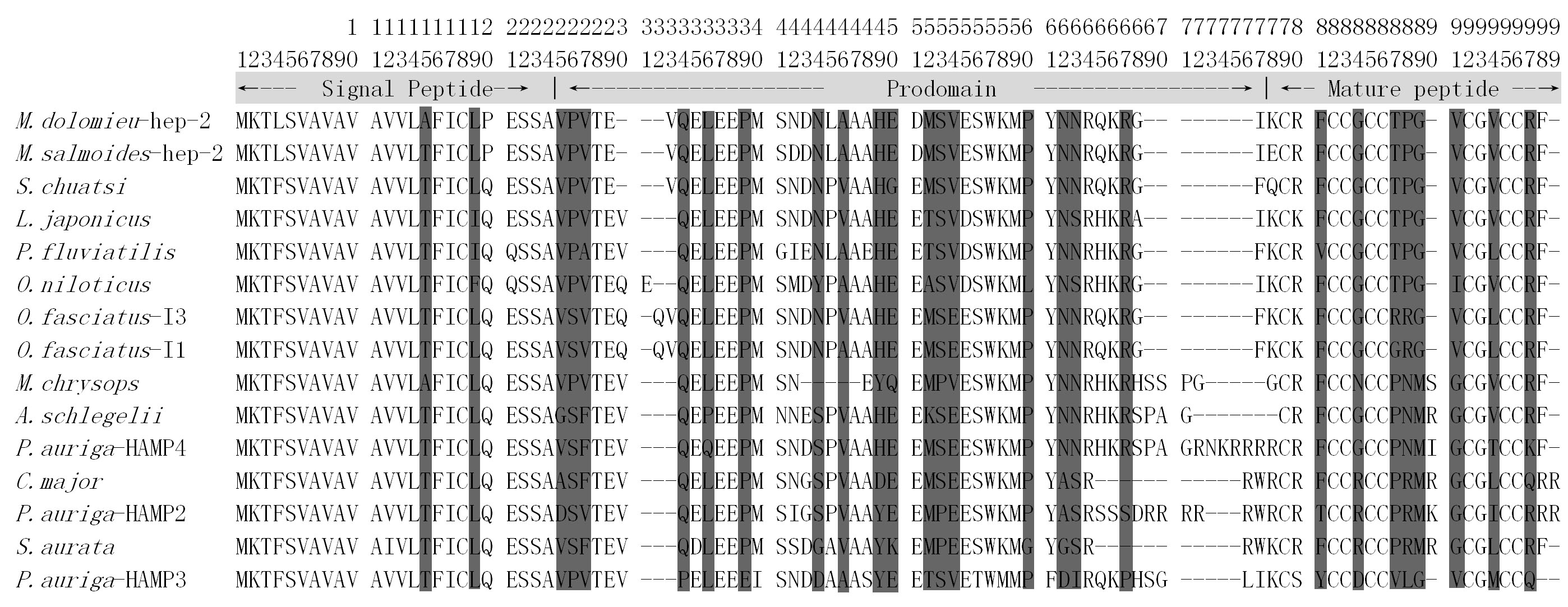

Supplement: Figure S3 — Amino acid sequence comparison among fish HAMP2 sequences. Positively selected sites identified using M8 model (Table 1) are shaded in black background. (JPG) [file pone.0035449.s003.jpg]
