# Supplementary material for: Miiuy Croaker Hepcidin Gene and Comparative Analyses Reveal Evidence for Positive Selection
Source: PLoS One. 2012 Apr 12;7(4):e35449. doi: 10.1371/journal.pone.0035449 (PMC3325200; doi:10.1371/journal.pone.0035449)
Supplement: Table S1 — Percent identity of amino acid (aa) sequences calculated versus the miiuy croaker HAMP sequence. (DOC) [file pone.0035449.s004.doc]

**Table S1** Percent identity of amino acid (aa) sequences calculated versus the miiuy croaker HAMP sequence

| Species | GenBank accession No. | Identity (%) |
| --- | --- | --- |
| *Micropterus dolomieu* hep-1 | EU502751 | 93.6 |
| *Micropterus salmoides* Hep-1 | EU502749 | 93.6 |
| *Notothenia angustata* | EU221589 | 90.9 |
| *Larimichthys crocea* | AM748024 | 94.6 |
| *Pagrus auriga* | AB440775 | 92.6 |
| *P. auriga* HAMP1 | AB440779 | 92.6 |
| *Lycodichthys dearborni* | EU221608 | 89.6 |
| *Scophthalmus maximus* | AY994074 | 89.6 |
| *Pseudopleuronectes americanus* | AW013026 | 86.2 |
| *Paralichthys olivaceus* | AY533022 | 84.8 |
| *Monopterus albus* | FJ594996 | 86.5 |
| *Micropterus dolomieu* hep-2 | EU502752 | 74.1 |
| *M. salmoides* hep-2 | EU502750 | 73.4 |
| *Siniperca chuatsi* | FJ876150 | 75.1 |
| *Lateolabrax japonicus* | AY604195 | 74.4 |
| *Perca fluviatilis* | EF602303 | 73.1 |
| *Oreochromis niloticus* | DQ388036 | 69.4 |
| *Oplegnathus fasciatus* isoform 3 | EU809942 | 73.4 |
| *O. fasciatus* isoform 1 | EU809944 | 72.7 |
| *Morone chrysops* | AF394246 | 71.0 |
| *Acanthopagrus schlegelii* | AY669377 | 72.4 |
| *Pagrus auriga* HAMP4 | AB440782 | 72.4 |
| *Chrysophrys major* | AY452732 | 70.4 |
| *P. auriga* HAMP2 | AB440780 | 66.3 |
| *Sparus aurata* | EF625901 | 72.1 |
| *P. auriga* HAMP3 | AB440781 | 70.7 |
| Norway cat | AF344185 | 50.5 |
| house mouse | AF297664 | 35.7 |
| dog | AY899807 | 27.3 |
| Pig | AF516143 | 41.1 |
| human | BC020612 | 25.3 |
