# Supplementary material for: Miiuy Croaker Hepcidin Gene and Comparative Analyses Reveal Evidence for Positive Selection
Source: PLoS One. 2012 Apr 12;7(4):e35449. doi: 10.1371/journal.pone.0035449 (PMC3325200; doi:10.1371/journal.pone.0035449)
Supplement: Table S2 — Genomic organization of hepcidin genes of different fish species. (DOC) [file pone.0035449.s005.doc]

**Table S2** Genomic organization of hepcidin genes of different fish species

| Species | Exon 1 (bp) | Intron 1 (bp) | Exon 2 (bp) | Intron 2 (bp) | Exon 3 (bp) | GenBank |
| --- | --- | --- | --- | --- | --- | --- |
| *Miichthys miiuy* | 87 | 100 | 78 | 167 | 104 | HQ889846 |
| *Pagrus major* | 87 | 104 | 78 | 144 | 93 | AY452733 |
| *Monopterus albus* | 87 | 99 | 78 | 160 | 108 | FJ594996 |
| *Paralichthys olivaceus* | 87 | 100 | 78 | 133 | 105 | AY623818 |
| *Micropterus dolomieu* Hep-1 | 87 | 100 | 78 | 161 | 108 | EU502751 |
| *Micropterus dolomieu* Hep-2 | 87 | 111 | 78 | 132 | 96 | EU502752 |
| *Micropterus salmoides* Hep-1 | 87 | 100 | 78 | 161 | 108 | EU502749 |
| *Micropterus salmoides* Hep-2 | 87 | 87 | 78 | 132 | 96 | EU502750 |
| *Scophthalmus maximus* | 87 | 114 | 78 | 172 | 108 | AY994075 |
| *Pagrus auriga* HAMP1 | 87 | 100 | 78 | 166 | 108 | AB440779 |
| *Pagrus auriga* HAMP2 | 87 | 104 | 78 | 144 | 117 | AB440780 |
| *Pagrus auriga* HAMP3 | 87 | 93 | 78 | 166 | 102 | AB440781 |
| *Pagrus auriga* HAMP4 | 87 | 104 | 78 | 118 | 123 | AB440784 |
| *Morone chrysops* | 87 | 99 | 63 | 191 | 108 | AF394245 |
| *Oplegnathus fasciatus* I1 | 87 | 99 | 84 | 153 | 96 | EU809944 |
| *Sparus aurata* | 87 | 104 | 78 | 125 | 90 | EF625901 |
| *Perca fluviatilis* | 87 | 105 | 78 | 166 | 96 | EF602303 |
| *Oreochromis niloticus* | 87 | 95 | 81 | 143 | 96 | DQ388036 |
